# Draft Genome Sequence of *Rhizoctonia solani* Anastomosis Group 1 Subgroup 1A Strain 1802/KB Isolated from Rice

**DOI:** 10.1128/genomeA.01188-17

**Published:** 2017-10-26

**Authors:** Kalaivani Nadarajah, Nurhani Mat Razali, Boon Huat Cheah, Nur Syafiqah Sahruna, Ismanizan Ismail, Madhura Tathode, Kiran Bankar

**Affiliations:** a School of Environmental and Natural Resource Sciences, Faculty of Science and Technology, Universiti Kebangsaan Malaysia, Bangi, Selangor, Malaysia; b School of Biosciences and Biotechnology, Faculty of Science and Technology, Universiti Kebangsaan Malaysia, Bangi, Selangor, Malaysia; c Bionivid Technology Private Limited, Kasturi Nagar, Bangalore, India

## Abstract

Sheath blight, caused by *Rhizoctonia solani* anastomosis group 1 subgroup 1A (AG1-1A), is one of the most devastating rice diseases worldwide. Here, we report the draft genome sequence of *R. solani* AG1-1A strain 1802/KB isolated from a popular Malaysian rice variety. To the best of our knowledge, this is the second reported representative genome from AG1-1A.

## GENOME ANNOUNCEMENT

*Rhizoctonia solani* Kühn (teleomorph: *Thanatephorus cucumeris* [A. B. Frank] Donk) is a destructive soilborne plant-pathogenic basidiomycete that causes diseases on a wide range of plant species (1). *R. solani* is a multinucleated heterokaryon containing significant heterozygosity within single cells (2). Based on hyphal anastomosis reactions, *R. solani* strains have been classified into 14 reproductively incompatible anastomosis groups (AGs), although little is known about the genetic determinants of this recognition process (3). Variable host range has been observed between AGs; for instance, AG8 was reported as a devastating broad-host-range pathogen causing diseases of wheat, barley, canola, and legumes (4), while AG1 subgroup 1A (AG1-1A) is capable of causing sheath blight infection in rice (5).

Sheath blight caused by *R. solani* AG1-1A is the second most devastating disease of rice after rice blast (6). Currently, there is only one reported genome of an *R. solani* AG1-1A isolate from China (5). Here, we report the draft genome sequence of a Malaysian *R. solani* strain, 1802/KB (GenBank accession number KF312465), which was isolated from the diseased rice tissues of MR219 variety on paddy fields (7). MR219 is a popular rice variety in Malaysia, yet it is reported to be highly susceptible to sheath blight (8). *R. solani* 1802/KB, as shown by our previous disease symptom screening, is a more virulent strain of sheath blight than another Malaysian *R. solani* isolate, 1801/UPM (GenBank accession number KF312464) (8).

Genomic sequencing was performed on an Illumina MiSeq platform with 2 × 300-bp paired-end modules. High-quality (HQ) reads with ≥Q30 were selected from raw data using NGS QC toolkit version 2.3.3 (9). The HQ paired-end reads were assembled with Velvet version 1.2.10 (10) by selecting a k-mer of 35 to generate primary contigs. These primary contigs were then subjected to scaffolding and anchoring against the nearest reference genome of *R. solani* AG1-1A (GenBank assembly accession number GCA_000334115.1) to generate a draft genome using MeDuSa (11). Scaffolds with lengths shorter than 500 bp were excluded to reduce the fragmented nature of the assembly.

The final draft of 28,929,909 bp was generated and assembled into 1,517 scaffolds (*N*_50_, 420,418 bp), with 46.99% G+C content. A total of 10,037 predicted genes were found to be present in the genome assembly, of which 7,072 genes could be mapped to the fungal nonredundant databases (NRDB). Comparatively, 10,489 open reading frames (ORFs) were reported in the pioneering *R. solani* AG1-1A isolate from China (5). Quality assessment of our genome assembly and annotation completeness based on evolutionarily informed expectations of gene content using Benchmarking Universal Single-Copy Orthologs (BUSCO) (12) indicated that 986 out of a total of 1,335 BUSCO groups from Basidiomycota are present as single-copy genes, indicating a fairly high degree of genome completeness. Furthermore, 2 rRNA- (1 from 18S and 1 from 28S rRNA) and 117 tRNA-coding genes were predicted in the assembly. This assembly provides an important foundation for future comparative analyses that aim to discover the genetic components responsible for the varied host range, disease symptoms, genome plasticity, and evolution observed across AGs.

### Accession number(s).

This whole-genome shotgun project has been deposited at DDBJ/ENA/GenBank under the accession number MRJL00000000. The version described in this paper is version MRJL02000000.
